# Regular gastroscopy and colonoscopy during the evaluation of urachal cancer: do we really need them?

**DOI:** 10.1186/s12885-023-11531-9

**Published:** 2023-11-27

**Authors:** Ming-rui Luo, Tao-nong Cai, Jiang-li Lu, Zhen-hua Liu, Sheng-jie Guo, Zhuo-wei Liu, Kai Yao, Zi-ke Qin, Yun-lin Ye

**Affiliations:** 1 Department of Urology, Sun Yat-sen University Cancer Center, Guangzhou, 510060 P. R. China; 2 State Key Laboratory of Oncology in South China, Sun Yat-sen University Cancer Center, Guangzhou, 510060 P. R. China; 3 Collaborative Innovation Center for Cancer Medicine, Guangzhou, 510060 P.R. China

**Keywords:** Urachal cancer, Bladder adenocarcinoma, Gastroscopy, Colonoscopy, Imaging examination

## Abstract

**Purpose:**

Urachal cancer is similar to gastrointestinal adenocarcinoma in histology, and gastroscopy/colonoscopy is often administered during perioperative evaluation. However, gastroscopy and colonoscopy have corresponding disadvantages. This study discusses whether gastroscopy/colonoscopy is truly necessary for patients with urachal cancer.

**Patients and methods:**

A total of 166 bladder adenocarcinoma cases diagnosed at Sun Yat-sen University Cancer Center were retrospectively reviewed and divided into two groups (urachal cancer and nonurachal cancer), and perioperative evaluations were retrieved.

**Results:**

There were 78 patients with urachal cancer, the median age was 48 years, and 59 were male. Perioperative gastroscopy/colonoscopy revealed 5 intestinal polyps and 1 adenoma during these evaluations, and no primary gastrointestinal cancer was found. Meanwhile, preoperative imaging evaluation did not detect significant gastrointestinal lesions. For 88 patients with nonurachal cancer, including primary bladder adenocarcinoma and metastatic tumors from gastrointestinal cancer, the median age was 56 years, and 64 were male. Preoperative imaging evaluation demonstrated 36 cases of gastrointestinal lesions, and 32 were confirmed by gastroscopy/colonoscopy; the other 4 were negative. Another 4 cases of colon cancer were detected by regular colonoscopy for suspected primary bladder adenocarcinoma. In all, 35 cases of colon cancer and 1 case of gastric cancer were identified by endoscopic examination. The diagnostic consistency of imaging and gastrointestinal endoscopy was favorable (P < 0.001), and the negative predictive value and diagnostic efficiency of imaging were 96.9% and 94.6%, respectively.

**Conclusions:**

The vast majority of gastrointestinal cancer cases can be identified by assessment of the patient’s clinical symptoms, meticulous physical examination, and imaging evaluation. We recommend that gastroscopy/colonoscopy only be applied to patients with urachal cancer when the above examinations are positive.

Urachal cancer, a rare nonurothelial tumor of the bladder that has a poor prognosis, often occurs at the junction of the urachal ligament and bladder dome [1–3]. Because the histology of urachal cancer is similar to that of gastrointestinal adenocarcinoma, gastroscopy and colonoscopy are often administered during the perioperative evaluation to exclude metastasis from primary gastrointestinal cancer [4]. However, some clinicians believe that metastasis of gastrointestinal tumors does not frequently present as isolated urachal tumors. In clinical practice, most gastroscopy and colonoscopy examinations are negative for patients with suspected bladder adenocarcinoma when imaging does not demonstrate gastrointestinal involvement. In addition, the majority of adenocarcinomas at other sites can be excluded by a detailed medical history inquiry and physical examination, and basically all gastrointestinal tumors involving the bladder would show evidence in the imaging examination [5]. Therefore, we considered that regular gastroscopy and colonoscopy might not be necessary for the perioperative evaluation of all patients suspected to have urachal cancer.

To explore this hypothesis, patients with urachal tumors or bladder adenocarcinoma treated at Sun Yat-sen University Cancer Center during the last 20 years were retrospectively collected, and gastroscopy and colonoscopy results were reviewed.

## Materials and methods

### Patients

**Fig. 1:**
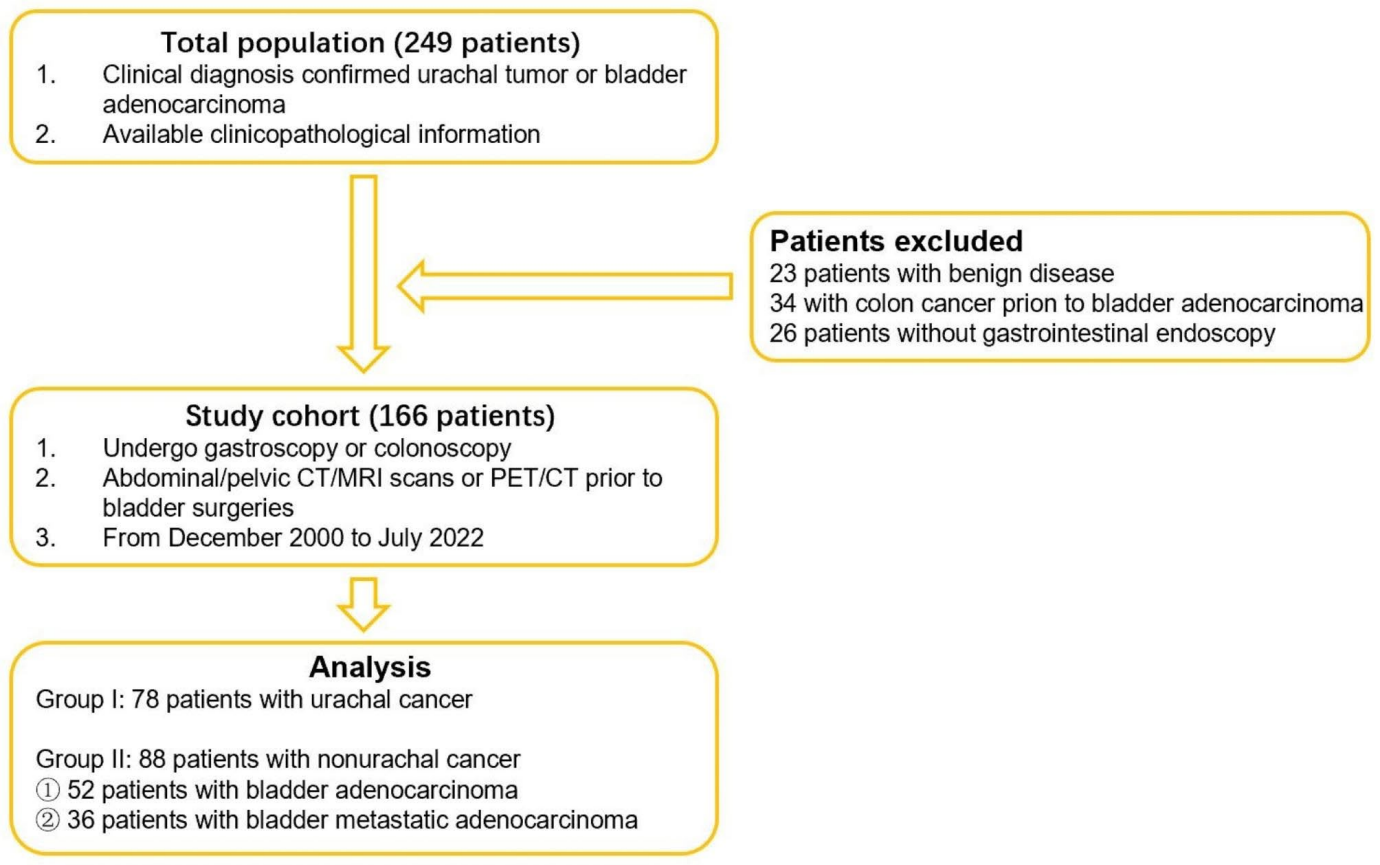
We have enrolled 166 patients and divided into two groups. The Group I were 78 patients with urachal cancer, and 88 patients in Group II had nonurachal cancer. Among the nonurachal cancer, there are 52 patients with primary bladder adenocarcinoma and 36 patients with metastatic/invasive adenocarcinoma from gastrointestinal

With the approval of the ethics committees of Sun Yat-sen University Cancer Center (No. GZR2018-053), 249 patients with a diagnosis of urachal tumor or bladder adenocarcinoma who were referred to our cancer center were enrolled. Under review of pathological results, 23 patients with benign disease, 34 with colon cancer prior to bladder adenocarcinoma and 26 without gastrointestinal endoscopy were excluded. Then, the remaining 166 patients were divided into two groups (78 patients with urachal cancer and 88 patients with nonurachal cancer), and perioperative evaluations were retrieved (Fig. 1). Among 88 patients with nonurachal cancer, there are 36 patients with secondary adenocarcinoma of urinary bladder from gastrointestinal adenocarcinoma and 52 patients with primary bladder adenocarcinoma. These 52 patients had tumors located on the any part of bladder other than the top or front wall, the pathological results indicated adenocarcinoma, gastroscopic and enteroscopic examinations showed no signs of gastrointestinal tumor, and no history of gastrointestinal disease.

All patients had abdominal/pelvic CT/MRI scans or PET/CT prior to bladder surgeries, chest CT scans were completed to exclude lung cancer, and tumor markers associated with gastrointestinal cancers, such as CEA, CA19-9 and CA125, were tested regularly before bladder surgery or when adenocarcinoma was confirmed. Gastroscopy and colonoscopy were administered during the perioperative period, including 88 cases before curative bladder operation (radical cystectomy or extended partial cystectomy) and 78 cases after it. During follow-up, abdominal CT/MR or PET/CT and related serum tumor markers were administered every 3–6 months, and gastroscopy and colonoscopy were administered when suspected gastric/colon cancer was indicated.

## Results

In all, there were 78 patients with urachal cancer, the median age was 48 years old, and 59 were male. Among the 88 patients with nonurachal cancer, the median age was 56 years old, and 64 were male, including 52 with primary bladder adenocarcinoma and 36 with metastatic tumors from gastrointestinal cancer. Markers including CEA and CA19-9 were not differentiated between these two groups (Table 1).

**Table 1:** Clinical characteristics between two groups

	Urachal cancer (%)	Non-urachal cancer (%)	P value
Gender (Male)	59 (75.6)	64 (72.7)	0.7
Age (> 60 years)	16 (20.5)	30 (34.1)	0.05
CEA (> 5 ng/ml)	20 (25.6)	25 (28.4)	0.7
CA19-9 (> 35 U/ml)	9 (11.5)	14 (15.9)	0.4
Gastrointestinal involvement in imaging	1 (1.3)	36 (40.9)	<0.01

Of the 78 patients with urachal cancer, 75 underwent extended partial cystectomy, 2 underwent radical cystectomy and urinary diversion, and the other patient with distant metastasis underwent diagnostic biopsy. According to the Sheldon staging system, 63 cases were stage III, and 15 cases were stage IV. At a median follow-up of 36 months, 17 patients died from urachal cancer. Perioperative gastroscopy and colonoscopy were administered in 67 patients, and colonoscopy alone was administered in 11 patients. Five intestinal polyps and 1 adenoma were detected during these evaluations, and no primary gastrointestinal cancer was found in 78 patients. Meanwhile, preoperative imaging evaluations did not reveal significant gastrointestinal lesions, except for one case with suspected intestinal involvement in the imaging examination that was derived from a history of appendicitis and adhered to the right anterior bladder wall.

All of the 88 patients with nonurachal adenocarcinoma of the bladder underwent TURBT or biopsy as initial treatment. Both gastroscopy and colonoscopy were administered to 74 patients, and only colonoscopy was performed in 14 patients. Preoperative imaging evaluation demonstrated 36 cases of gastrointestinal lesions, 32 of which were confirmed by gastroscopy and colonoscopy; the other 4 were negative. Another 4 cases of colon cancer were detected by regular colonoscopy for suspected primary bladder adenocarcinoma. In all, 35 cases of colon cancer and 1 case of gastric cancer were identified by endoscopic examination. Accordingly, 52 patients were diagnosed with primary adenocarcinoma, 30 underwent radical cystectomy, 10 underwent TURBT, and 12 underwent partial cystectomy as major treatments. At a median follow-up of 38 months ( interquartile range: 16–71 months), 21 died from cancer, and none exhibited gastrointestinal cancer up to the last follow-up. For 36 patients with bladder metastatic adenocarcinoma, palliative surgery including bladder tumor resection was administered in 25 patients, and chemotherapy and/or other system therapies were performed for all cases. At a median follow-up of 24 months (interquartile range: 14–45 months), 22 patients died from cancer.

As shown in Table 2, the diagnostic consistency of imaging and gastrointestinal endoscopy was favorable (P < 0.001), and the diagnostic value of imaging was as follows: sensitivity-88.9%, specificity-96.2%, positive predictive value-86.5%, negative predictive value-96.9%, and diagnostic efficiency-94.6%. For patients with no gastrointestinal lesions on imaging, gastrointestinal endoscopy detected only 4 cases of primary gastric or colon cancers. Especially for patients with urachal cancer, which has a typical location in the bladder dome, no additional gastrointestinal cancer was detected during the perioperative evaluation and follow-up.

**Table 2:** Diagnostic efficiency of two methods

			Gastrointestinal involvement in imaging		
Gastroscopy/colonoscopy examination			Negative (%)	Positive (%)	In all (%)
	All patients	Negative	125 (96.9)	5 (13.5)	130 (78.3)
	(n = 166)	Positive	4 (3.1)	32 (86.5)	36 (21.7)
		All	129 (100)	37 (100)	166 (100)
	Patients with urachal cancer	Negative	77 (100)	1 (100)	78 (100)
	(n = 78)	Positive	0 (0)	0 (0)	0 (0)
		All	77 (100)	1 (100)	78 (100)
	Patients with nonurachal cancer	Negative	48 (92.3)	4 (11.1)	52 (59.1)
	(n = 88)	Positive	4 (7.7)	32 (88.9)	36 (40.9)
		All	52 (100)	36 (100)	88 (100)

## Discussion

Most urachal cancers are adenocarcinomas that are rare and potentially considered to be metastases, and primary cancer in other organs should be excluded [3, 6, 7]. Gastroscopy and colonoscopy are regularly administered during the perioperative evaluation to confirm whether there is primary gastrointestinal cancer. In our clinical practice, gastroscopy and colonoscopy are regularly administered to most urachal cancer patients, similar to patients with other bladder adenocarcinomas, to exclude primary gastrointestinal cancer, which is the most common adenocarcinoma. However, it is extremely rare that the anterior wall or dome of the bladder is first involved in metastatic gastric cancer or colon cancer [8]. Across the guidelines and consensuses regarding urachal cancer, no definite statement regarding gastroscopy or colonoscopy has been confirmed, although they have recommended excluding primary cancers from other common organs [3, 6].

In this cohort, no gastrointestinal cancer was detected in patients with suspected urachal cancer, and imaging examination seemed to have a diagnostic efficacy comparative to gastrointestinal endoscopy for patients with urachal cancer, which had typical imaging features: location of the tumor in the dome or anterior wall and epicenter of the tumor in the bladder wall. The negative predictive value and diagnostic efficiency were nearly 95%. In one patient with suspected involvement of the ascending colon, chronic appendicitis involved the dome of the bladder. For 52 patients who were diagnosed with primary nonurachal adenocarcinoma, regular gastroscopy and colonoscopy detected 4 cases of colon cancer. These results suggest that gastroscopy and colonoscopy might not be necessary for all patients with suspected urachal carcinoma.

As early as 2012, a urachal adenocarcinoma study proposed that an additional workup looking for an alternate occult primary is not typically necessary but should be considered if the patient’s history or physical examination suggests a possible alternate primary source [9, 10]. First, isolated metastases from gastrointestinal adenocarcinoma to the bladder are very infrequent; second, imaging examinations have high sensitivity; furthermore, although endoscopy is the gold standard for diagnosing cancers of the gastrointestinal tract, this approach is invasive and results in discomfort and many complications (such as colonoscopic perforations and bleeding), and the price is relatively expensive [5, 11]. Gastroscopy/colonoscopy as a tool to exclude metastases in regular follow-up may not be the best choice.

**Fig. 2:**
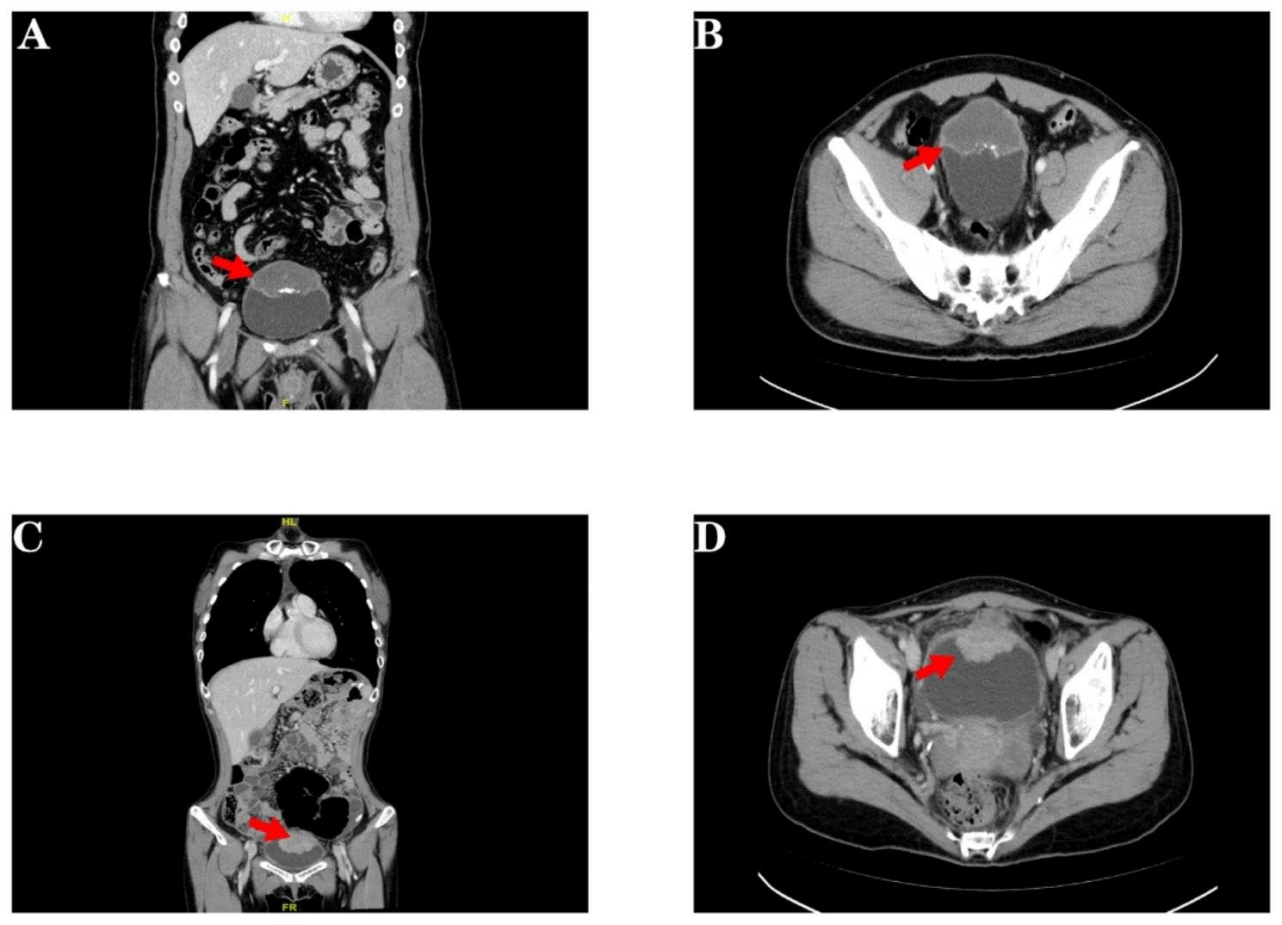
**A** and **B** present the imaging of a patient with primary urachal cancer. There was a 3.0*2.7 cm saccular outpouching in the anterior superior bladder wall, and urachal diverticulum was considered. The bladder superior wall showed a 6.5*4.8 cm mass, which was diagnosed as urachal cancer. **C** and **D** present the imaging of a gastric adenocarcinoma patient with bladder metastasis. Uneven thickening of the bladder wall can be observed, approximately 3.1 cm at its thickest part. The patient had significant gastrointestinal symptoms (abdominal pain and black stools) and several other sites of metastasis on imaging

Patient history and physical examination are essential components in the diagnosis of gastrointestinal tract tumors. Some of the common symptoms are abdominal pain, abdominal distention, and bloody stool, and the specific symptoms of colorectal cancer are changes in bowel habits and intestinal obstruction [12–14]. Abdominal examination and digital rectal examination are important tools in the diagnosis of gastrointestinal tract tumors. In addition, with the development of imaging examinations, most primary gastrointestinal cancers can be detected during perioperative imaging evaluations (Fig. 2) [15, 16]. A recent study of ovarian cancer also supports this view. This article revealed that gastroscopy/colonoscopy had limited power to differentiate metastatic ovarian tumors when compared to imaging. Imaging and gastroscopy/colonoscopy had similar diagnostic efficiencies in detecting gastrointestinal cancer in patients with suspected ovarian cancer [17, 18]. In contrast to ovarian tumors, for which it is difficult to obtain sufficient specimens for pathological diagnosis, bladder tumors can yield enough specimen tissue, this can help the clinicians to improve diagnostic accuracy. Therefore, compare with ovarian cancer, it can reduce the reliance on gastrointestinal endoscopy, the importance of endoscopy is decline, only if the imaging examination reveals suspected gastrointestinal tract tumors.

With development of imaging examinations, most gastrointestinal cancer could be detected during perioperative imaging evaluation. For gastric adenocarcinomas, the particular CT finding is loss of the multilayered pattern and thickening of the gastric wall. Focal thickening greater than 5 mm on CT in a distended stomach indicates a neoplastic lesion. For colorectal adenocarcinoma, the CT features can be presented as focal and irregular thickening of the wall, infiltration of the surrounding fatty tissue, serosal irregularity and with lymphadenopathy.

To the best of our knowledge, this is the first investigation of urachal carcinoma and gastroscopy/colonoscopy. Here, the limited number of cases and retrospective analysis were inevitable limitations. Because of the rarity of urachal carcinoma, this sample is the best we could obtain, and large-population, multicenter research is needed.

## Conclusion

In contrast to other bladder adenocarcinomas, urachal carcinoma is rarely associated with gastrointestinal cancer. The vast majority of gastrointestinal cancers can be detected by assessment of the patient’s clinical symptoms, meticulous physical examination and imaging evaluation. We recommend that gastroscopy/colonoscopy should only be applied for patients with urachal carcinoma when the above examinations are positive.

## Data Availability

The authenticity of this article has been validated by uploading the key raw data onto the Research Data Deposit public platform (www.researchdata.org.cn), with the application RDD number as (RDDA2023888789).
